# The Relationship between Psychological Disability and Religious Practice and Coping Strategies in Caregivers of Children with Traumatic Brain Injury in Pakistani Population

**DOI:** 10.3390/healthcare10112158

**Published:** 2022-10-28

**Authors:** Ayesha, Saboor Ahmad, Shazadi Saba, Muhammad Kashif, Danish Ali Khan, Absarul Haque, Muhammad Imran Naseer, Adel M Abuzenadah, Anwar M. Hashem, Shafiq Ur Rehman

**Affiliations:** 1 College of Physical Therapy, Northwest Institute of Health Sciences, Peshawar 25100, Pakistan; 2 King Fahd Medical Research Center, King Abdulaziz University, Jeddah 21859, Saudi Arabia; 3 Department of Medical Laboratory Sciences, Faculty of Applied Medical Sciences, King Abdulaziz University, Jeddah 21859, Saudi Arabia; 4 Center of Excellence in Genomic Medicine Research, King Abdulaziz University, Jeddah 21589, Saudi Arabia; 5 Vaccines and Immunotherapy Unit, King Fahad Medical Research Center, King, Jeddah 21589, Saudi Arabia

**Keywords:** traumatic brain injury, religious practice, psychological distress, coping strategies, caregiver, children

## Abstract

Background: Traumatic brain injury (TBI) is a serious issue and a leading cause of death and disability worldwide. Caregivers of TBI patients experience psychological distress and a variety of social and financial issues. The present study aims to investigate the caregiver’s burden and the factors that influence this burden. Furthermore, the present study will find out the association of religious practice, religious coping relations and psychological distress among caregivers of children affected with TBI. Methods: A cross-sectional survey was conducted on 302 caregivers of children with TBI using Duke University Religion Index (DURL) for religious practice. General Health Questionaire-12 (GHQ-12) was used for anxiety and depression and Brief Religious Coping Scale (RCOPE) was used for coping strategies. The caregivers were conveniently chosen from different regions of Khyber Pakhtunkhwa province and data was collected from different tertiary care hospitals in Peshawar. Results: Forty-nine (49) % of caregivers score ≥ 3 on GHQ suffer from psychological distress with a Mean of 20.957 ± 4.175). Positive coping methods were mostly used by caregivers than negative coping have a low level of distress with a Mean Positive Coping (P-COPE ) of 6.93 ± 0.41, Mean of Negative Coping (N-COPE) 0.486 ± 1.023. In religious practice, caregivers mostly participate in Organized Reliogious Activities (ORA) or some Non-Organized Reliogious Activities (NORA) with a Mean ORA of 4.20 ± 1.27, and NORA Mean of 4.17 ± 1.37 used by the caregivers. Coping methods were related to Caregiver psychological distress (GHQ-12 and P-COPE co-relation scores are (ρ −0.022, p b 0.05); GHQ-12 scores and N-COPE (ρ + 0.221=, p b 0.001). There is a negative correlation between GHQ 12 and PCOPE, while GHQ12 is positively correlated with NCOPE. Conclusion: According to this study, there is a significant association between religious coping methods, religious practice, and psychological distress among caregivers of children with traumatic brain injury.

## 1. Introduction

Traumatic brain injury (TBI) is a brain injury, caused by out-sided force to the head that affects brain function [1]. TBI is caused by a focal gunshot wound or diffuse brain injuries like shaken baby syndrome or both types. Symptoms depend on the area of the lesion, and the extent of brain damage [2]. According to the Centers for Disease Control and Prevention (CDC), the most common causes of TBI in children aged 1 to 18 years old are falls (50.2%), struck by/against them (24.8%), automobile collisions (6.8%), assault on a person (2.9%) and other/unknown (15.3%) mechanisms of TBI in children TBI is a general health concern that can result in short- or long-term memory impairment. TBI causes psychological discomfort and financial burden all around the world [3]. Caregivers suffer from economic burdens as well as psychological discomfort during the TBI treatment process. The caregiver experiences feelings of hopelessness, frustration, impotence, despair, depression, grief, and a desire to quit. TBI has a significant impact on patients, their families, communities, and healthcare systems [4].

For both the survivor and the survivor’s family, the healing process after a TBI can be lengthy and challenging. During a hospital stay, caregivers may neglect their home and work responsibilities, and they may have difficulty returning to their pre-injury activities [4]. They may feel a greater sense of responsibility to provide physical, financial, and emotional support than they are used to. TBI is the leading cause of death in children in the age group of 0–5 years and 15–19 years [5]. In addition, 145,000 children aged 1–19 years are living with long-term changes in behavior, physical disability and cognitive impairments following TBI [6]. The findings from a systematic review revealed that siblings experience psychological suffering and worse family functioning following TBI in children including behavioural problems in their brother and sisters and absence of perceived social support. The findings largely support that there is a need to grow and evaluate support interventions to improve siblings’ adjustment to the effects of TBI [7]. Although evidence of efficacy is lacking, however, a number of tactics have been defined in the literature for siblings support in the context of TBI [8,9].

People react differently to different types of events and have varying levels of sensitivity. Caregivers who are experiencing psychological discomfort may have a different reaction. Cognitive evaluation and coping mechanisms, according to Lazarus and Folkman’s model, have a major impact on the quality and severity of a person’s stress reaction in a stressful event. Religion helps in stress management by increasing the occurrence of pleasant sensations and decreasing the likelihood of negative thoughts like depression, stress, and suicidal thoughts [10]. According to Kaplan et al, religion has a distinctive role in three ways when coping with various disorders: First people engage in a religious event, secondly, religion helps people to cope with stressful events, and lastly, religion is essential in people’s lives to cope [11]. 

Numerous studies have been conducted to examine how religious participation influences caregivers’ distress levels when their children suffer from long-lasting illnesses such as cancer, thalassemia, or cystic fibrosis. Caregivers, who pray, attend public religious services and study religious scriptures had lower stages of anxiety [12,13,14]. Some caregivers cope with their circumstances by seeking God’s comfort [11]. As a result, religious beliefs and practices can alter people’s perceptions of terrible occurrences and diminish unpleasant sensations, either directly or indirectly, while also encouraging pleasant emotions and the development of human values [15]. 

Religious coping stratagies are categorized into negative religious coping and positive religious coping [16]. There are more hopeful ways to deal with positive religious relationships that include spiritual help, forgiveness, shared religious conflicts, spiritual connections, and compassionate religious reconsideration Negative coping is a stressful approach to dealing with issues like spiritual misery and blaming God. Positive religious survival methods are more often used by people caring for children with chronic conditions than negative ones [17,18,19].

According to our knowledge, no study has been conducted on “the relationship between religious practice and psychological distress and coping strategies among caregivers of children affected with TBI. The diverse types of religious coping have had different effects on how people react to catastrophic life experiences. This study aims to look at different religious coping techniques in predicting coping strategies in a group of TBI child caregivers.

## 2. Materials and Methods

A cross-sectional survey was conducted at Northwest General Hospital & Research Centre (NWGH), Khyber Teaching Hospital (KTH), Hayatabad Medical Complex (HMC) Peshawar, the leading health care providing hospitals in Khyber Pakhtunkhwa province. The data was collected after approval from the ethical committee (Approval ID; NWIHS-EC/0028-0021) of the Northwest Institute of Health Sciences, Peshawar, Pakistan and the declaration of Helsinki was followed. The population for the study was caregivers of children with TBI at the above mention hospitals. Non-Probability convenience sampling was used. Inclusion criteria were considered (Caregivers of TBI patients, Patients aged 1 to 18 years). The data was collected through General Health Questionaire-12 (GHQ-12) (for behavioral changes and psychological distress), for religious practice the Duke University Religion Index (DUREL) and Brief Religious Coping Scale (Brief RCOPE) were used for coping. Participants meeting the inclusion criteria were selected for the study. To all participants, the purpose of the study was explained and informed consent was obtained in written form. All the willing participants were briefed about the purpose and procedure of this study. We provided a questionnaire in Urdu (translated through a faculty member of the department of Engish, University of Peshawar, who were native Urdu speakers) format for those who are not able to understand the questionnaire in English. For those participants who were illiterate, the questionnaires were administered by the researcher through a face-to-face interview, while others filled them through self-administration. The data collector verbally asked questions. The investigator was there to assist in case of difficulty in understanding. Prior to the survey, a short introduction was given to participants. Those participants who permitted us were screened by the inclusion (Caregivers of TBI patients, children aged 1 to 18 years) and exclusion criteria (known cases of Acute TBI patients, patients with any physical issues, diabetic neuropathy, amputation, mentally retarded patients and congenital brain disorder) in these tertiary care hospitals. To analyze the data SPSS (Statistical Package for Social Sciences) version 20 was used. Using descriptive statistics, Demographic data were described in tables. The links between, religious coping techniques, religious practice, and psychological distress were investigated using Spearman’s rank-order correlation. 

## 3. Results

The probable sample size was 302, 95% confidence interval, however, 310 questionnaires were given to Caregivers who met inclusion criteria throughout the sampling period. Three hundred and two caregivers agreed to take part and completed the questionnaires, in total, 79 % (*n* = 239) were male and 21% (*n* = 63) were female. In terms of religion, 99.4 % (*n* = 99) were Muslim, while only 0.6 % (*n* = 1) were Christian Table 1. 

### 3.1. Religious Practice, Religious Coping Methods and Psychological Distress

The psychological distress Mean or average score was 20.957 ± 4.175. Almost all of the caregivers (*n* = 302) were depressed, with a GHQ score of 3 or above. The mean(M) score for Organized Religious activities (ORA), and caregivers’ religious practice was 5.738 ± 0.7343, suggesting a high level of attendance at public religious services or meetings; the sample had a high ORA score. The mean for Non-Organized Religious Activities (NORA) was 4.410 ± 1.452, and the participants practiced it. The mean score for Intrinsic religiosity (IR) caregivers’ religious practice was 14.367 ± 1.270, with IR ranging from 3 to 15; high IR for the majority, indicating strong religious motivation and faith. In terms of the coping sample score high on P-COPE, the mean P-COPE was 6.93 ± 0.418. The sample used negative religious coping methods N-COPE mean was 0.4868 ± 1.023. whereas the range show number of question in the questionnaire. Table 2 shows the mean, Std. Deviation, Ranges of religious practices, psychological distress and religious coping of the participants are presented in Table 2.

### 3.2. Relationship between Psychological Distress and Religious Practice

The Spearman rank-order correlations between religious coping methods (N-COPE and P-COPE), religious practices sub-scales (NORA, ORA, and IR), and psychological distress (GHQ-12). ORA and GHQ-12 scores had a weak negative correlation (rs (df) = −0.020, p b 0.05), ORA is inversely related to GHQ 12 when a person deviates from ORA then his/her distress level will ultimately decrease. NORA and GHQ-12 scores had a weak positive connection (rs (df) = 0.094, p b 0.05), with an increase in NORA linked with high distress. IR and GHQ-12 had a negative connection (rs (df) = −0.045, p N 0.05). The connection between religious coping strategies and psychological discomfort-COPE and GHQ-12 scores had a weak negative correlation (rs (df) = −0.022, p b 0.05); increase P-COPE was linked with reduced distress level. The positive connection between GHQ-12 scores and N-COPE (rs (df) = 0.221, p b 0.05), with increased N-COPE scores being associated with increased distress (Table 3).

### 3.3. Spearman Rank-Order Correlations between Psychological Distress and Religious Practice and Religious Coping Method 

Table 4 shows the Spearman rank-order correlations between religious coping methods (N-COPE and P-COPE), religious practices sub-scales (NORA, ORA, and IR), and psychological distress (GHQ-12). The co-relation GHQ12 with NORA, ORA, IR, NCOPE and PCOPE has already been discussed in Table 3.

Whereas in Table 4, the second row shows the inter and intra-co-relation between questioners. There is a weak negative inter-co-relation between ORA and GHQ 12 (−0.020; *p* = 0.728). When a person deviates to ORA then his distress level will ultimately decrease. A moderate positive intra-co-relation between ORA and NORA (ρ 0.234) is observed with high significance (*p* < 0.000). There is a weak positive intra-co-relation between ORA and IR, which is highly significant (ρ = 0.175; *p* = 0.002). There is a weak positive intercorrelation between ORA and PCOPE (ρ = 0.125; *p* = 0.030). There is a moderate negative intercorrelation between ORA and NCOPE (ρ = −0.363; *p* = 0.000) which is highly significant. The third row shows the inter and intra-co-relation between questioners. There is a weak positive inter correlation between NORA and GHQ 12 (ρ = 0.094; *p* = 0.102) showing no significance. There is moderate positive intra-co-relation between NORA and ORA (ρ 0.234, *p* = 0.000) indicating highly significance. There is a moderate positive intra-co-relation between NORA and IR, which is highly significant (ρ = 0.298; *p* = 0.000). There is a weak positive inter-co-relation between NORA and PCOPE with no significance (ρ = 0.049; *p* = 0.399). There is a moderate negative intercorrelation between NORA and NCOPE with no significance (ρ = −0.094; *p* = 0.104). The fourth row shows the inter and intra-co-relation between IR, GHQ12, ORA, NORA PCOPE and NCOPE, There is a weak negative inter-co-relation between IR and GHQ 12 (ρ = −0.045; *p* = 0.434) which is non-significant. There is a weak positive intra-co-relation between IR and ORA (ρ 0.175). here, *p*-value 0.002 indicates high significance. There is moderate positive intra-co-relation between IR and NORA, which is highly significant (ρ = 0.298, *p* = 0.000). There is a weak positive inter co-relation between IR and PCOPE (ρ = 0.131, *p* = 0.022) indicating significance. There is a weak negative intercorrelation between IR and NCOPE (ρ = −0.173, *p* = 0.003) which is significant. The fifth row shows the inter and intra-co-relation between PCOPE, IR, GHQ12, ORA, NORA and NCOPE, There is a weak negative inter co-relation between PCOPE and GHQ 12 with no significance (ρ = −0.022; *p* = 0.709). There is a weak positive intercorrelation between PCOPE and ORA (ρ 0.125; *p* = 0.709) with no Significance. There is a moderate positive intercorrelation between PCOPE and NORA, which is non-significant (ρ = 0.049, *p* = 0.399). There is a weak positive intercorrelation between PCOPE and IR (ρ = 0.131, *p* = 0.022) which shows that the Correlation is significant at the 0.05 level (2-tailed). There is moderate negative intra-co-relation between PCOPE and NCOPE (ρ = −0.296, *p* = 0.00) which is highly significant. The last row shows the inter and intra-co-relation between NCOPE, IR, GHQ12, ORA, NORA and PCOPE, There is moderate negative inter co-relation between NCOPE and GHQ 12 with high significance (ρ = −0.22; *p* = 0.000). NCOPE and ORA showed moderate positive inter co-relation and high significance (ρ −0.363, *p* = 0.000). There is a weak negative inter co-relation between NCOPE and NORA, which is non–significant (ρ = −0.049, *p* = 0.104). There is a weak negative inter correlation between NCOPE and IR (ρ = −0.173, *p* = 0.003) which shows that the Correlation is significant at the 0.05 level (2-tailed). There is moderate negative intra-co-relation between NCOPE and PCOPE (ρ = −0.296, *p* = 0.000) which is highly significant.

## 4. Discussion

In this research, nearly all of the caregivers caring for a child suffering from traumatic brain injury (*n* = 302) reported psychological problems, with GHQ-12 ratings of 3 or higher. This finding is comparable to the findings of Shaligram et al. (2007), who found that about half (50%) of their observers had known psychiatric problems and scored > 3 on the GHQ-12 [20]. In a study conducted by Ali et al., 2012, out of 40, 27 (67.5%) parents of children who experience hypochromic anemia, reported psychological problems and scored > 3 on the GHQ-12 [21]. This discrepancy could be attributed to the short size of the sample or demographic variables. On the other side, the majority of parents (58%) scored < 3 on the GHQ-12, suggesting a decreased distress level or none at all, possibly due to acclimating to the standard therapies. The high ORA (M = 5.738, *n* = 302) and NORA (4.410, *n* = 302) scores suggested that caregivers were often engaged in religious practices. The average mean scores revealed an increased utilization of NORA and ORA, showing that caregivers take part in group religious activities and private religious practices. In contrast, Hexem et al. (2011) discovered that parents prayed and read religious scriptures more frequently, although not in a regular manner. Attending places of worship, according to Hexem et al is a sort of community aid in which friends offer support and strength to sick kids by praying for them. The parent’s average intrinsic religiosity score indicates that they have a high level of IR, strong faith, and find inspiration in religious instruction. Parents who did not regularly join group religious services sensed a connection with God [13]. This is supported by previous studies conducted by Atkin and Ahmad et al. (2000) in which parents agreed that religion is important [14]. The P-COPE mean score of religious coping was 6933 indicating that parents used P-COPE more than negative methods. The finding is constant with earlier research demonstrating that parents select positive religious coping mechanisms [11,14,17]. Furthermore, the use of P-COPE may be due to their belief and faith in God which strengthens them to cope with it [12]. The connection between psychological distress and religious practice even though there is a weak negative connection between ORA and psychological distress in caregivers [rs (df) = −0.020, p b 0.05], the findings indicate that increased ORA will decrease psychological discomfort. This data suggests that those caregivers, who are experiencing a great level of emotional distress are trying to go to community religious services to connect and receive support from the community. Previous research has also indicated that religious activity is related to emotional support from God [13,14,22]. On the other hand, the study findings showed that caregiver psychological discomfort and NORA are negatively associated; caregivers’ who regularly engaged in NORA (e.g reading religious scriptures, meditation, or prayer) had decreased distress. Because the link was weak, and the outcome may happen by coincidence. Longitudinal research would help to explain the association between psychological discomfort and religious activities in the future.

No statistically significant relationship between psychological distress and IR, implying that neither low nor high religious motivation scores were linked to caregivers’ psychological discomfort or may be due to individual perceptions in the intensity of psychological suffering felt by caregivers who are subjected to the pressures [17]. Psychological distress is determined by how the individual assessed the circumstance based on religious instruction. An individual’s stress response during stressful conditions is determined by their coping technique and intellectual evaluation [23]. The connection between psychological discomfort and religious coping strategies, Positive religious coping mechanisms were found to be associated with decreased levels of psychological discomfort in this study. Thombre et al. (2010) backed up this conclusion, claiming that religious coping was linked to higher levels of positive life-change perceptions. Caregivers who use negative coping methods have increased psychological distress than caregivers who used P-COPE. This is similar to the research of advanced cancer patients (aged 21 and older) and their non-related caregivers (N = 162) that specified that negative coping practices were connected with anxiety disorders and increased the risk of major depression [24,25]. Negative religious coping mechanisms show the effort that results from a damaged relationship with God. A negative view of life, and a sense of hostility from a religious community. As a result, nurses have a hard time paying attention to and being aware of their patients’ religious coping styles during ordinary nursing care. On admission, nurses might conduct a cultural and spiritual assessment to gather previous information to assist caregivers who require religious support. Screening parents and caregivers for religious coping mechanisms could be useful in identifying people who are at risk of psychological distress and giving spiritual support to help them regain control of their circumstances [25,26].

### 4.1. Limitations 

There are various restrictions. As convenience sampling was used and there were chances of occurrence of selection biases. There might have been some caregivers experiencing more psychological discomfort and they may have been overlooked. Random sampling can be used to avoid bias. Because this study relied on questionnaires and required participants to react at that moment, some caregivers may have struggled to focus due to the urge to comfort their distressed child. The investigators discovered that caregivers may answer quickly, to finish the questionnaire, leading to recall bias. As a result, the caregiver should be given enough time and space to complete the questionnaire, especially when their children are relaxing. There was also the possibility of self-report bias, which impeded the accuracy of the caregiver’s information, reducing generalizability. A longitudinal study is recommended in the future to evaluate the relationship between religious coping methods and psychological suffering among caregivers caring for children with traumatic brain injury. Finally, because the researcher was in charge of all recruitment, the caregiver may have felt obligated to participate in the study. Note that the translation of Questionare from English to Urdu language was not subject to study verification.

### 4.2. Practice Implication 

Caregivers in this study challenged a variety of problems that impacted their psychological well-being. Even though the data showed a modest association, there are several practical implications. When caregivers suffer from stress, religious coping approaches are offered to them as non-religious coping methods to cope with these stressful events. Nurses can connect parents in need with other religious colleagues. Caregivers gain control and confidence over their psychological well-being contributes to better child health outcomes. Religious coping might be an option for assisting caregivers in coping with their suffering. As a result, when delivering nursing care, nurses should respect each culture. Furthermore, continual nursing education on this topic is critical in strengthening the nurses’ comprehension of their coping techniques. Moreover, nurses may work with a religious front-runner like an imam, holy man, pastor, or other religious crops to help careers manage stress and to offer nurses a better understanding of religious beliefs. Finally, caregivers who do not have religious beliefs should be taken into account. These caregivers may require therapy, coping skills training, or membership in a TBI support group.

## 5. Conclusions

In the present study, we for the first time found in Pakistani poulation that caregivers (*n* = 302) caring for children with traumatic brain injury endure psychological hardship. We noted an incidence of anxiety and depressive symptoms in caregivers. The caregivers take part in group religious activities as well as private religious practices which correlates with decreased psychological discomfort while managing the TBI children. This data also suggests that those caregivers who are experiencing a great level of emotional distress are trying to go to community religious services to connect and receive support from the community. They relied on positive religious coping strategies more than negative religious coping strategies. Non-Organized, and Organized Religious Activities, and negative and positive religious coping mechanisms had a strong relationship with caregivers’ psychological suffering.

## Figures and Tables

**Table 1:** Showing characteristics, frequencies, and percentages.

Variable	Characteristics	Frequency	Percentage
Gender	Male Female Total	239 63 302	79.1% 20.9% 100.0%

**Table 2:** Caregivers ’ Mean, Std. Deviation, psychological distress, religious practices, and religious coping.

Variables	Total No	M ± SD	Ranges
**Psychological distress (GHQ-12)**	302		
≤3 scores ≥3 scores		20.957 ± 4.175	0–12
**Religious practice (DUREL)**			
NORA ORA IR		4.410 ± 1.452 5.738 ± 0.734 14.367 ± 1.270	1–6 1–6 1–6
**Religious coping Method**			
N-COPE P-COPE		0.486 ± 1.023 6.933 ± 0.418	0–7 0–7

**Table 3:** Correlations among religious practice, religious coping methods, and psychological distress.

Variables	Values	GHQ-12 Scores
**Religious Practices**		
ORA NORA IR	ρ p ρ p ρ p	−0.020 0.728 0.094 0.102 −0.045 0.434
**Religious Coping Method**		
P-COPE N-COPE	ρ p ρ p	−0.022 0.709 0.221 0.000 *

* Correlation is significant at the 0.05 level (2-tailed). *p* < 0.05 was statistically significant.

**Table 4:** Correlations among, religious coping methods, religious practice and psychological Distress.

			GHQ12	ORA	NORA	IR	PCOPE	NCOPE
**Spearman’s rho**	**GHQ12**	Correlation Coefficient	1.000	−0.020	0.094	−0.045	−0.022	0.221
		Sig.(2 tailed) *p* value		0.728	0.102	0.434	0.709	0.000 *
		N	302	302	302	302	302	302
	**ORA**	Correlation Coefficient	−0.020	1.000	0.234	0.175	0.125 *	−0.363
		Sig. (2-tailed) *p* Value	0.728		0.000 *	0.002 *	0.030 *	0.000 *
		N	302	302	302	302	302	302
	**NORA**	Correlation Coefficient	0.094	0.234	1.000	0.298	0.049	−0.094
		Sig.(2 tailed) *p* value	0.102	0.000 *		0.000 *	0.399	0.104
		N	302	302	302	302	302	302
	**IR**	Correlation Coefficient	−0.045	0.175	0.298	1.000	0.131 *	−0.173
		Sig.(2 tailed) *p* value	0.434	0.002 *	0.000 *		0.022 *	0.003 *
		N	302	302	302	302	302	302
	**PCOPE**	Correlation Coefficient	−0.022	0.125 *	0.049	0.131 *	1.000	−0.296
		Sig.(2 tailed) *p* value	0.709	0.030 *	0.399	0.022 *		0.000 *
		N	302	302	302	302	302	302
	**NCOPE**	Correlation Coefficient	0.221	−0.363	−0.094	−0.173	−0.296	1.000
		Sig.(2 tailed) *p* value	0.000 *	0.000 *	0.104	0.003 *	0.000 *	.
		N	302	302	302	302	302	302

* Correlation is significant at the 0.05 level (2-tailed). *p* < 0.05 was statistically significant.

## Data Availability

The data will be available upon the author’s request to the corresponding author.
